# New Drugs, Appliances, and Things Medical

**Published:** 1891-06-06

**Authors:** 


### NEW DRUGS, APPLIANCES, AND THINGS MEDICAL.

[All preparations, appliances, novelties, etc., of which a notice is desired, should be sent for The Editor, to care of The Manager, 140, Strand, London, W.C.]

### THE "HALF MINUTE" CLINICAL THERMOMETER.

Messrs. Hudson, Crosby Square, E.C., have sent us a specimen of their registering thermometer. We find it registers in 35 seconds, and that compared with a Kew standard instrument it stands the usual tests well. We have also carried and used it for a week in ordinary practice, where again it has given satisfaction. When we add that the price is 3s. 6d., in a metal case, we can sum up its value by saying it answers its purpose admirably, saving both time and money.

### PLASTERS AND BANDAGES.

(ST. DALMAS, LEICESTER.)

We again have pleasure in noticing these excellent articles. Mr. St. Dalmas has not only continued the manufacture of his well-known plasters and bandages, but has added considerably to their number and variety since we last noticed them. Among them we find adhesive plaster on holland and on fine calico for general purposes; also spread on pink flesh-coloured cambric, both in roller and in ribbon form. This latter is a very elegant and convenient adjunct to the "dressings" drawer, requiring no second pair of hands to hold the roll whilst a piece is being cut off to suit the case. The same plaster is also put up in six-inch widths in canisters. Then there are corn and other special plasters, all well made, and serving excellently for the purposes intended. Next we notice absorbent pads mounted on stout linen, the edges of which are made adhesive. These are always ready, and most useful. A good specimen of belladonna plaster spread on moleskin, and a really "protective" perforated plaster bring the specimens submitted to our notice to a close. This last is composed of a backing of good warm red flannel, on which is spread the adhesive material, the whole being closely perforated to enable the excretions of the skin to escape. Beside the plasters, the proprietor has sent us some of the St. Dalmas' bandages. These are beautifully soft, and made so that the edges do not become unravelled, a great saving of time and patience, as those who have to do much bandaging know. In conclusion, we can state that the "Leicester" goods are well made, strong, and answer their purposes admirably. We note that their manufacturer is quite alive to the needs of the day, and is always ready to produce any of his goods in the forms demanded by the increasing knowledge and experience of the surgical world.

